# Coping with COVID-19: The Strategies Adapted by Pakistani Students to Overcome Implications

**DOI:** 10.3390/ijerph18041799

**Published:** 2021-02-12

**Authors:** Gul Muhammad Baloch, Kamilah Kamaludin, Karuthan Chinna, Sheela Sundarasen, Mohammad Nurunnabi, Heba Bakr Khoshaim, Syed Far Abid Hossain, Areej Al Sukayt, Laareb Gul Baloch

**Affiliations:** 1 School of Medicine, Faculty of Health and Medical Sciences, Taylor’s University, No. 1, Jalan Taylors, Subang Jaya 47500, Selangor, Malaysia; karuthan.chinna@taylors.edu.my; 2 Department of Accounting, Prince Sultan University, P.O. Box 66833, Riyadh 11586, Saudi Arabia; kkamaludin@psu.edu.sa (K.K.); ssundarasen@psu.edu.sa (S.S.); mnurunnabi@psu.edu.sa (M.N.); asukayt@psu.edu.sa (A.A.S.); 3 Deanship of Educational Services, Prince Sultan University, P.O. Box 66833, Riyadh 11586, Saudi Arabia; hkhoshaim@psu.edu.sa; 4 College of Business Administration, International University of Business Agriculture and Technology, 4 Embankment Drive Road, Sector-10, Uttara Model Town, Dhaka 1230, Bangladesh; sfa_hossain@yahoo.com; 5 School of Medicine, University of Leeds, Leeds LS2 9JT, UK; um19lgb@leeds.ac.uk

**Keywords:** coping strategies, anxiety, adaptive/maladaptive, problem-focused coping, students, mental health, COVID-19, Pakistan

## Abstract

COVID-19 has speedily immersed the globe with 72+ million cases and 1.64 million deaths, in a span of around one year, disturbing and deteriorating almost every sphere of life. This study investigates how students in Pakistan have coped with the COVID-19. Zung’s self-rating anxiety scale (SAS) was used for measuring anxiety and the coping strategies were measured on four strategies i.e., seeking social support, humanitarian, acceptance, and mental disengagement. Among 494 respondents, 61% were females and 77.3% of the students were in the age group of 19–25 years. The study findings indicate that approximately 41 percent of students are experiencing some level of anxiety, including 16% with severe to extreme levels. Seeking social support seemed to be the least preferred coping strategy and that female students seek social support, humanitarian, and acceptance coping strategies more than males. Students used both emotion-based and problem-based coping strategies. The variables of gender, age, ethnicity, level and type of study, and living arrangement of the students were associated with usage of coping strategies. Findings showing that students do not prefer to seek social support. The study outcomes will provide basic data for university policies in Pakistan and the other countries with same cultural contexts to design and place better mental health provisions for students.

## 1. Introduction

### 1.1. The Beginnings and Current Scenario: Global and Pakistan

The diseases transmitted to humans through animals are known as zoonotic diseases. Jones [1] suggests that “majority of emerging infectious diseases (71.8%) originate in wildlife and are increasing significantly over time.” CDC [2] estimates that “more than 6 out of 10 known infectious diseases in people can be spread from animals, and 3 out of every 4 new or emerging infectious diseases in people come from animals.” WHO [3] asserts that “globally, about one billion cases of illness and millions of deaths occur every year from zoonoses.” The number of confirmed cases of COVID-19, a zoonotic disease, peaked to 72,851,747 and caused 1,643,339 deaths globally as of 17 December 2020, in a span of about one year since its first report [4]. Director General of WHO declared this as a “Public Health Emergency of International Concern” (PHEIC) on 30th January 2020; the term COVID-19 was coined on 11 February 2020. The first case of COVID-19 in Pakistan was detected on 25th February and the first reported death on 29th March 2020 [5]. COVID-19 stepped into Pakistan via the returning pilgrims from religious gatherings/visits to Saudi Arabia, Iran, Malaysia, and Indonesia [6]. The returning Pakistani students from China were also the source of spread of COVID-19 in Pakistan. The current number of confirmed cases in Pakistan, as of 17 December, is 445,977, with deaths at 9010 [7].

Mak et al. [8] mention in their study about SARS that “SARS can be regarded as a mental health catastrophe” which resulted in “post-traumatic stress disorder and depressive disorders.” A very recent study by Haider [9] also indicates that pandemics like COVID-19 increase stress levels and that “it is possible that people may begin to experience transient mild to moderate depressive symptoms.” Being an integral part of society, students are undoubtedly affected by the epidemics. A study by Zeng [10] found that and overwhelming majority of the student had suffered from “fear, helplessness, worry and depression even though none of them had SARS,” and almost the “same number of students (59.6%) felt an impact of SARS on their college life.” Yet another study done by Wang & Zhao [11] regarding the impacts of COVID-19 found “higher anxiety” levels in university students.

To contain the COVID-19 epidemic, the government of Pakistan had announced country-wide closure of all schools, colleges, and universities on 13th March 2020 [5]. The educational institutions re-opened from 15 September 2020 with instructions to observe SOPs. However, this closure of the educational institutions also closed “a source of many students to cope with numerous personal and familial issues” [12]. This same study also found that “young students who had exposure to the COVID-19 pandemic are more vulnerable to predispose of mental health issues.” In a study done by Salman [13] with 1134 respondent students from Pakistani universities, it was found that COVID-19 have “significant adverse impact on student’s mental health.” A study by Balkhi [14] found “a higher tendency for graduates to fear for the safety of their health, even at home (*p* < 0.01).” This study was done in Karachi, the first city in Pakistan to report the COVID-19 cases.

### 1.2. Coping and Coping Strategies

Carver [15] defines coping as “efforts to prevent or diminish threat, harm, and loss, or to reduce the distress that is often associated with those experiences.” Thus, coping is “basic process integral to adaptation and survival, as it depicts how people detect, appraise, deal with, and learn from stressful encounters” [16]. Coping involves use of different strategies and techniques in order to manage the situation. Coping is “problem-focused” when “directed at reducing the threats and losses of the illness,” and it is “emotion-focused” when “directed as reducing the negative emotional consequences” [17]. The individual attempts to change the source of stress or redefine the hazardous situation through acceptance, resignation, or other things.

Coping strategies are defined as “as efforts to regulate emotions, behaviors, cognitions, psychophysiology, and environmental aspects in response to the stress of everyday events” [18]. It is also found that adopting the coping strategy largely determines how individuals experience anxiety [19], to help an individual being shielded. Making communication, avoidance, and exercise are some of the strategies that are used [20]. Students in Malaysia adopted strategies of exercise, praying, counseling, and meditation including yoga and tai chi [21].

### 1.3. Aims of the Study

Individuals engage in coping strategies to manage the stress, specifically when confronting a significant life change or traumatic event. Brooks [22] reported psychological impacts due to quarantine like “post-traumatic stress symptoms, irritability, insomnia, poor concentration, confusion, depression, low mood, emotional exhaustion and anger.” This study investigates how students in Pakistan have coped with the COVID-19 epidemic and the ensuing measures of lockdown that have affected the educational institution as well.

## 2. Materials and Methods

### 2.1. The Study

A self-administered validated questionnaire was used that was initially pilot tested. Subsequently, the final version of the questionnaire was sent via WhatsApp and email to university students, using Google Forms. Respondents from 50 universities and colleges participated in the study from all the four provinces/states of Pakistan. The major institutions were Bahria University, Baqai Medical University, Bolan University of Medical and Health Sciences, Dow University of Health Sciences, National University of Computer and Emerging Sciences, Federal Medical and Dental College, IBA, International Islamic University, Iqra University, Jinnah Medical & Dental College, Jinnah Sindh Medical University, Lahore Garrison University, Liaquat University of Medical & Health Sciences, Mehran University of Engineering & Technology, Peoples Medical University, Peoples Medical University of Health Sciences for Women, Quaid-i-Azam University, SMBB Medical University, University of Sindh, University of Karachi SZABIST, and Ziauddin University. Data were collected during the lockdown period from 1 June to 15 June 2020. The questionnaire was unnamed, and informed consent was sought. Ethical approval was obtained from the institutional review boards (IRB) of the partaking universities.

### 2.2. The Research Instrument

The research instrument included basic personal and demographic data, and Zung’s self-rating anxiety scale (SAS) [23]. The questionnaire comprised questions about the students’ general demographics, assessment of anxiety and questions on how the students coped with the psychological impact of COVID-19 and the effect of lockdown. The questionnaire was content-validated by an expert panel of psychologists and pilot-tested among 20 university and college students. In this survey, the students’ anxiety levels were calculated using Zung’s SAS, a validated 20-item self-report instrument. The instrument employs a Likert-type scale of 1–4 (1 = Never or very rarely; 2 = Sometimes; 3 = Often; 4 = Very often or always). Questions 1–5 characterized the emotional pointers of anxiety, whereas questions 6–20 inquired about physical anxiety symptoms. Scores ranged from 1 to 4 points per question. Scores >50 denoted psychological anxiety, while scores of 50–59 indicated mild anxiety, 60–69 moderate anxiety, and >70 severe anxiety. Based on Zung [23], the anxiety scale in “physical and mental health scores” has a test-retest reliability of 0.70 and criterion-related validity of 0.60. As coping strategies used may differ from chronic conditions and physiological impact in the midst of a pandemic, questions were designed adapting questions from previous studies. In this study, the use of four coping strategies—seek social support, acceptance, mental disengagement, and humanitarian—was assessed. Seeking social support refers to emotional or instrumental support from family and friends that provide stress-related interpersonal aid. An example of the questions: “During COVID-19 and lockdown, I talked to someone about how I was feeling.” Acceptance is the adaptation to unchangeable negative events by maintaining the individual’s psychological well-being and capacity to act. An example of questions asked: “About COVID-19 and lockdown, I learned to live with it.” Mental disengagement involves directing attention and effort toward alleviating negative emotions by engaging in substitute activities to keep one’s mind from ongoing stressors. An example of questions asked: “To take my mind away from COVID-19 and lockdown, I watched TV.” Lastly, humanitarian coping involves the initiatives taken by one in helping others in psychosocial despair. An example of questions asked: “During COVID-19 and lockdown, I called/ texted/videoed my friends to give them emotional support.” The questionnaire was content-validated by an expert panel of psychologists. The items were measured on a scale of 1–4; 1 = never/rarely, 2 = sometimes, 3 = often, and 4 = very often/always. In the pilot study, the Cronbach’s alpha values were a 0.931, 0.923, 0.683, and 0.796 for the items in “seek social support” (4 items), “acceptance” (4 items), “mental disengagement” (2 items), and “humanitarian” (3 items) constructs, respectively. In the final analysis, for each coping strategy, the mean scores for the respective items were computed; higher scores implied a higher level of use.

### 2.3. Data Analysis

The data were analyzed using SPSS 22.0 software. Quantitative variables were summarized as means and standard deviations, while qualitative variables were described as frequencies and percentages. For the data analysis, *t*-test, analysis of variance (ANOVA) and multivariate analysis of variance (MANOVA) procedures were used. For ANOVA and MANOVA, the *p*-values were adjusted using Tukey’s multiple comparisons procedure. The level of significance was 0.05 for all tests.

### 2.4. Ethical Approval

The ethical approval was obtained Prince Sultan University Saudi Arabia (PSU IRB-2020-04-0038, Dated May 7, 2020), and Khairpur Medical College, Pakistan (KMC/RERC/29, Dated 30 May 2020).

## 3. Results

### 3.1. Demographic Analysis

A total of 510 responses were received. After data cleaning, 494 responses were found to be usable. From the 494 respondents, 60.9% were females and 39.1% were males. A percentage of 77.3% of the students were between 19–25 years, 13.5% were above 26 years, and the remaining 9.1% were below 18 years old. Most of the respondents were studying health sciences (39.1%) followed by social sciences (28.5%), sciences (19.8%), management (6.5%), and engineering (6.1%). Almost three-quarters of the respondents were pursuing a professional program, whereas 20% were at the undergraduate level and 5% were at the postgraduate level. Notably, 37.9% of the respondents were enrolled in their first year, 19.6% in their second year, and 9.5% were in their third year. The remaining 33% were enrolled in their fourth year and beyond. Majority of the students (69%) responded that they had transitioned towards virtual learning during the pandemic. In terms of accommodation, 91% of the students were staying at their family homes, 6.6% were staying outside campus, and only 2.4% were living in college residencies.

### 3.2. Levels of Anxiety among College Students during the Epidemic

In the 20-item Zung’s SAS, each item is scored on a scale of 1 to 4. The summary scores for the 20 items are shown in Table 1. For each respondent, the total anxiety score was obtained by adding the responses for the 20 items. The total score ranged from 20 to 80. The scores were then converted to an “Anxiety Index” with values ranging from 25 to 100. According to Zung [23], an Anxiety Index <45 indicates “Anxiety within normal range,” a value in the range of 45–59 indicates “Mild to moderate anxiety,” a value in the range of 60–74 indicates “marked to severe anxiety,” and values ≥75 indicates “Most extreme anxiety.” 

Among the respondents in the sample, 125 (25.3%), 45 (9.1%), and 34 (6.9%) experienced mild to moderate, marked to severe, and most extreme levels of anxiety, respectively. For further analysis, cases with marked to severe anxiety and most extreme anxiety were grouped together and named as “Severe to Extreme” level of anxiety; Table 1.

### 3.3. Coping Strategies and Anxiety

This study aimed to know what strategies the students used in coping with anxiety during the COVID-19 pandemic. Four coping strategies, “Seek social support,” “Acceptance,” “Mental disengagement,” and “Humanitarian” were tested. The items were measured on a scale of 1 to 4; 1 = never/rarely, 2 = sometimes, 3 = often and 4 = very often/always. For each coping strategy, mean scores for respective items were computed, where higher scores implied higher level of use. The descriptive statistics for the coping strategies are shown in Table 2. Overall, the usage of all four strategies was moderate. The distributions were fairly normal (skewness <2, kurtosis <7). Overall, the students practiced more acceptance strategies and less seeking social support strategies.

The associations between coping strategies used and level of anxiety were tested using Analysis of variance (ANOVA) procedures. In the analyses, the variances were similar. The results are shown in Table 3.

Out of the four, only “Seek social support” and “Humanitarian” were significantly associated with level of anxiety. The usage of “Seek social support” coping strategy was higher in the “Severe to Extreme” anxiety group compared to the “Minimal to Moderate” and “Normal” groups. The usage of “Humanitarian” coping strategy was higher in the “Severe to Extreme” anxiety group compared to the “Normal” groups.

The following table is the summary from the post-host test. The pairwise differences are indicators using superscripts of a and b in Table 4.

### 3.4. Differences in Coping Strategies According to Students’ Demographic Characteristics

The differences in the usage of the four coping strategies were tested using Multivariate analysis of variance (MANOVA) procedure. The results are tabulated in Table 5.

The results in Table 5 indicate that female students seek social support, engaged in acceptance and humanitarian coping strategies more than male. The analysis also suggests that there was a significant difference in the usage of acceptance coping strategy by field of study. The usage of this strategy was higher among the students from engineering and management compared to those in social studies.

## 4. Discussion

This study examined the coping strategies adopted by university students in Pakistan due to psychological impacts of the COVID-19 pandemic. Findings indicate that approximately 41% of students are experiencing some level of anxiety. From these students, 16% are having severe to extreme anxiety levels, which is in line with earlier studies about students’ anxiety during the pandemic in other countries [24,25,26].

Four types of coping strategies i.e., acceptance, humanitarian, mental disengagement, and seeking social support were examined. Salin et al. [27] mention that most common emotion-focused coping method “adopted among the youth was acceptance.” Acceptance is “intended to disrupt the link between thoughts and behaviours to tolerate painful stimulation” [28]. The acceptance strategy involves a coping method that resolves into accepting the stressor in question. This pandemic is a health crisis that is beyond one’s control. Thus, students perceive that there is not anything much that can be done to overcome the pandemic other than to protect themselves from risk and exposure to the virus; thus, accepting the current situation. In our study, although usage of all four strategies were used moderately, students practiced more acceptance strategies (Mean 2.48 ± 0.79). This corroborates findings from earlier studies [29,30] that examined coping strategies during the SARS epidemic outbreak in China. Humanitarian is helping others for “physical and psychosocial well-being and actively providing compassionate and unconditional positive assistance” [31]. It is intended to “save lives, alleviate suffering and maintain human dignity during and after crises and disasters” [32]. Humanitarian coping action is found ranking second among the respondents (Mean 2.14 ± 0.71). Also known as problem-focused coping, trying to engage in doing something to alter the source of the stress, students shared food and were empathetic and supportive towards one another. The students experiencing severe to extreme anxiety level adopted acceptance (Mean 2.63 ± 0.72), followed by social support (Mean 2.28 ± 0.89) and humanitarian (Mean 2.28 ± 0.71) coping actions. 

The results of this study also indicated that the coping action involving seeking social support seemed to be the least preferred coping strategy (Mean 1.98 ± 0.77). American Psychological Association [33] defines social support as “the provision of assistance or comfort to others, typically to help them cope with biological, psychological, and social stressors.” Discussing social support, “contextual sensitivity” and approach is also important and useful, as argued by Williams [34]. While the students were actively engaged in extending support to others it did not appear that they themselves prefer seeking support to alleviate their own concerns about the pandemic. Nonetheless, students who are experiencing severe to extreme level of stress do seek social support more than others. By seeking social support from others, individuals reduce the perception of a potentially threatening situation like the pandemic as only stressful [35].

While others are also going through pandemic, this may be the underlying reason for this strategy adopted by the students to be the least preferred. Findings also show that female students seek social support (females: 2.06; males: 1.87), humanitarian (females: 2.23; males: 2.0), and acceptance (females: 2.56; males: 2.35) coping strategies more than males. Earlier studies have shown that females prefer maladaptive coping more than males [36]. This appears to be partially true as our results also indicate that females were also actively engaged in the humanitarian strategy, which is a problem-focused and adaptive coping more than their male counterpart. Thus, it appears that, be it an active or passive coping strategies, females are pro-active in both coping methods than their male counterparts. Arguably, in the condition of a pandemic outbreak, where the stressors are uncontrollable and unprecedented, even a maladaptive strategy is functional and adaptive [29,30], explaining the concurrent deployment of both methods of coping among female respondents [29].

Age-wise findings show that students of age bracket over 25 adopted more acceptance, humanitarian, and mental disengagement strategies than students of other age brackets (i.e., 18 and below, and 19–25 years). For seeking social support strategy, all the age groups had almost same mean values. 

Mental disengagement strategy, according to Traeger [37], is “employed to divert attention away from a stressor and toward other thoughts or behaviors that are unrelated to the stressor.” Although this strategy is employed to keep the mind away from the stressful event, Dijkstra [38], argues that it is “associated with lower perceived control, which in turn should be associated with lower psychological well-being.” Mental disengagement was the third preferred strategy adopted by the students (Mean 2.07 ± 0.55) and MANOVA shows almost same results for male (2.05 ± 0.59) and female (2.08 ± 0.52) students.

## 5. Conclusions

While many studies have explored the psychological effects of the pandemic to the society in general, limited studies focused on the mental health of the youth, particularly, the university students in Pakistan. This study examined how university students in Pakistan cope with the psychological impact of the COVID-19 pandemic. Based on Zung’s self-rating anxiety scale (SAS), 41% of the students are experiencing some level of anxiety, including 16% experiencing severe to extreme anxiety levels. Students’ coping mechanisms were evaluated based on four strategies, notably, seeking social support, humanitarian, acceptance, and mental disengagement. Seeking social support appears to be the least preferred coping method among the students. However, further analysis indicates that students who are experiencing severe to extreme level of anxiety sought social support more than others. Our findings also indicate that coping strategies vary based on students’ gender. Female students significantly seek social support, humanitarian, and acceptance coping strategies more than male students. Similarly, age, ethnicity, level, and type of study, and living arrangement of the students were also associated with usage of coping strategies.

## 6. Practical Applications

This study can help inform the government and policy makers about the youths’ preferred method of coping during a stressful situation like a pandemic. Students are young adults and at the vulnerable stage who get easily cajoled into opinions and views from the social media [11], which may invoke their sense of insecurity, fear, and helplessness during a public health crisis [39]. As such, it is imperative for government and policy makers to devise suitable policies to protect students.

## 7. Additionally

Universities and higher learning institutions shall conduct periodic assessment on students’ physical and mental health. While physical illness can be easily identified, mental health issues are more difficult to assess and resolve, particularly, in an environment where the students generally do not prefer seeking social support. Thus, learning institutions should assign trained mentors or advisors to review students’ emotional state during these critical times. Peer-to-peer counseling and chatgroups, moderated by a psychologist and an academic expert, can also help students to freely express their concerns and issues about their mental health and its impacts on their learning.

## 8. Limitations of the Study

Our study, which is based on cross-sectional design, is not intended to test for any causal relationships between students’ level of anxiety, coping strategies, and the overall psychological impact to the students. We suggest that future studies shall employ longitudinal design to test for these effects. Next, this study that uses self-reported questionnaire is subject to subjectivity and reliability issues. Finally, the limited sample size and convenience sampling approach may not reflect the bigger population of university students in Pakistan, which poses as another limitation to the study. 

## Figures and Tables

**Table 1:** Anxiety level based on Zung’s classification.

Anxiety	Frequency	Percentage	Anxiety	Frequency	Percentage
Normal	290	58.7	Normal	290	58.7
Mild to moderate	125	25.3	Minimal to Moderate	125	25.3
Marked to severe	45	9.1	Severe to Extreme	79	16.0
Most extreme	34	6.9			

**Table 2:** Descriptive statistics for coping strategies.

Coping Strategy	Mean ± S	Median	Skewness	Kurtosis
Seek social support	1.98 ± 0.77	2.00	0.724	0.055
Acceptance	2.48 ± 0.79	2.50	0.151	0.565
Mental disengagement	2.07 ± 0.55	2.00	0.464	0.501
Humanitarian	2.14 ± 0.71	2.00	0.399	0.278

**Table 3:** Anxiety level and coping strategies.

Coping Strategy	Anxiety Level	Mean ± S	F	*p*	Partial Eta-Square
Seek social support	Normal	1.87 ± 0.73 ^a^	9.683	*p* < 0.001	0.038
	Minimal to Moderate	2.05 ± 0.75 ^b^			
	Severe to Extreme	2.28 ± 0.89 ^a,b^			
Acceptance	Normal	2.42 ± 0.84	2.809	*p* = 0.061	0.011
	Minimal to Moderate	2.54 ± 0.67			
	Severe to Extreme	2.63 ± 0.72			
Mental disengagement	Normal	2.06 ± 0.57	0.527	*p* = 0.591	0.002
	Minimal to Moderate	2.06 ± 0.49			
	Severe to Extreme	2.13 ± 0.52			
Humanitarian	Normal	2.06 ± 0.70 ^a^	5.159	*p* = 0.006	0.021
	Minimal to Moderate	2.25 ± 0.71			
	Severe to Extreme	2.28 ± 0.71 ^a^			

^a,b^ pairwise differences S. standard deviation.

**Table 4:** Multiple Comparisons Tukey HSD.

Dependent Variable	(I) Anxiety.3	(J) Anxiety.3	Mean Difference (I–J)	Std. Error	Sig.
Seek.Soc.supp	Normal	Minimal to Moderate	−0.17186	0.08111	0.087
		Severe to Extreme	−0.41067 ^*^	0.09621	0.000
	Minimal to Moderate	Normal	0.17186	0.08111	0.087
		Severe to Extreme	−0.23881	0.10896	0.074
	Severe to Extreme	Normal	0.41067 ^*^	0.09621	0.000
		Minimal to Moderate	0.23881	0.10896	0.074
Humanitarian	Normal	Minimal to Moderate	−0.19016 ^*^	0.07509	0.031
		Severe to Extreme	−0.22753 ^*^	0.08907	0.029
	Minimal to Moderate	Normal	0.19016 ^*^	0.07509	0.031
		Severe to Extreme	−0.03737	0.10087	0.927
	Severe to Extreme	Normal	0.22753 ^*^	0.08907	0.029
		Minimal to Moderate	0.03737	0.10087	0.927

* The mean difference is significant at the 0.05 level.

**Table 5:** Results from Multivariate analysis of variance (MANOVA).

Variable	Seek Social Support	Acceptance	Mental Disengagement	Humanitarian
Gender	0.017	0.016	0.737	0.001
Female	2.06 ± 0.79 ^a^	2.56 ± 0.74 ^a^	2.08 ± 0.52	2.23 ± 0.71 ^a^
Male	1.87±0.73 ^a^	2.35 ± 0.84 ^a^	2.05 ± 0.59	2.00 ± 0.69 ^a^
Age	0.672	0.110	0.392	0.164
18 and below	1.89 ± 0.81	2.27 ± 0.81	2.08 ± 0.58	1.96 ± 0.64
19 to 25	2.01 ± 0.78	2.48 ± 0.77	2.05 ± 0.53	2.15 ± 0.73
>25	1.91 ± 0.68	2.65 ± 0.80	2.16 ± 0.59	2.23 ± 0.60
Field of study	0.364	<0.001	0.288	0.296
Engineering	2.09 ± 0.71	2.73 ± 0.85 ^a^	2.10 ± 0.47	2.23 ± 0.84
Health Sciences	2.04 ± 0.84	2.62 ± 0.79	2.11 ± 0.56	2.20 ± 0.71
Management	1.92 ± 0.76	2.73 ± 0.84 ^b^	2.22 ± 0.50	2.08 ± 0.65
Sciences	1.87 ± 0.72	2.37 ± 0.78	2.01 ± 0.62	2.05 ± 0.70
Social sciences	1.97 ± 0.73	2.26 ± 0.69^a,b^	2.02 ± 0.49	2.11 ± 0.68
Level of Study	0.820	0.164	0.211	0.910
Postgraduate	1.92 ± 0.68	2.53 ± 0.77	2.11 ± 0.52	2.17 ± 0.63
Professional	2.11 ± 0.90	2.84 ± 0.82	1.93 ± 0.64	2.22 ± 0.62
Undergraduate	1.99 ± 0.78	2.44 ± 0.78	2.07 ± 0.54	2.13 ± 0.73
Year of study	0.544	0.300	0.901	0.401
Year 1	1.93±0.75	2.42 ± 0.79	2.06 ± 0.56	2.05 ± 0.68
Year 2	1.95 ± 0.68	2.39 ± 0.75	2.08 ± 0.53	2.09 ± 0.72
Year 3	1.94 ± 0.77	2.48 ± 0.79	2.15 ± 0.51	2.23 ± 0.78
Year 4	2.01 ± 0.78	2.59 ± 0.76	2.05 ± 0.47	2.27 ± 0.69
Year 5	2.16 ± 0.91	2.62 ± 0.85	2.06 ± 0.65	2.22 ± 0.70
Accommodation	0.628	0.105	0.641	0.848
College residency/hostel	2.02 ± 0.60	2.60 ± 0.57	2.15 ± 0.66	2.14 ± 0.66
Family Home	1.98 ± 0.77	2.46 ± 0.78	2.07 ± 0.53	2.14 ± 0.70
Rented place	2.04 ± 0.86	2.70 ± 0.92	1.98 ± 0.64	2.13 ± 0.80

Value are mean ± standard error. The numbers in bold are p-values for the difference in the strategy used by the respective demographic characteristic. ^a,b^ pairwise differences.

## Data Availability

Not applicable.
